# Diet-induced obesity impairs refeeding responses and downregulates lateral septal GLP-1R in male rats: an effect reversed by weight-loss treatment

**DOI:** 10.3389/fphar.2026.1801283

**Published:** 2026-04-29

**Authors:** María José Covarrubias, Tatiana Dib, Jorge Escobar-Luna, Robinson D. Moreno, Daniela Cáceres-Vergara, Cristina Saldias, Georgina M. Renard, Patricio Iturriaga-Vásquez, Angélica P. Escobar, Gonzalo E. Torres, Ramón Sotomayor-Zárate

**Affiliations:** 1 Centro de Neurobiología y Fisiopatología Integrativa (CENFI), Instituto de Fisiología, Facultad de Ciencias, Universidad de Valparaíso, Valparaíso, Chile; 2 Programa de Doctorado en Ciencias Mención Neurociencia, Facultad de Ciencias, Universidad de Valparaíso, Valparaíso, Chile; 3 Programa de Magíster en Ciencias Biológicas Mención Neurociencias, Facultad de Ciencias, Universidad de Valparaíso, Valparaíso, Chile; 4 Centro de Investigación Biomédica y Aplicada (CIBAP), Escuela de Medicina, Facultad de Ciencias Médicas, Universidad de Santiago de Chile, Santiago, Chile; 5 Escuela de Medicina, Facultad de Medicina, Universidad de Valparaíso, Viña del Mar, Chile; 6 Departamento de Ciencias Químicas y Recursos Naturales, Facultad de Ingeniería y Ciencias, Universidad de la Frontera, Temuco, Chile; 7 Department of Molecular Pharmacology and Neuroscience, Stritch School of Medicine, Loyola University Chicago, Chicago, IL, United States

**Keywords:** GLP-1, GLP-1R, HFD, lateral septum, obesity

## Abstract

**Introduction:**

The lateral septum (LS) is a brain area involved in important physiological functions such as reward, stress response, and autonomic regulation. The LS is one of the brain areas with the highest expression of the glucagon-like peptide-1 receptor (GLP-1R), and pharmacological modulation of GLP-1R in the LS affects food intake. However, the relationship between obesity development and LS dysregulation of the GLP-1/GLP-1R system has been poorly studied.

**Methods:**

We aimed to investigate whether chronic exposure to a high-fat diet (HFD) from weaning to postnatal day (PND) 62 affects the LS GLP-1 system, LS neurotransmitter content, and post-fasting refeeding behavior in rats. Furthermore, we evaluated LS GLP-1R levels and refeeding behavior after pharmacological (phentermine, an amphetamine derivative) and dietary (switch to control diet) interventions.

**Results:**

Our results show that HFD decreases LS GLP-1R protein levels in male rats, while receptor gene expression increases in female rats. Otherwise, chronic exposure to HFD increases LS glutamate content only in female rats. Treatment with phentermine (30 mg/kg/day) and a control diet reduces body weight and fat tissues in obese rats of both sexes. However, in these animals, the re-exposure to fasting resulted in a marked preference for consuming HFD, an effect not observed in obese rats only exposed to a control diet. Finally, LS GLP-1R levels were normalized in obese male rats treated with phentermine plus a control diet or only with a control diet.

**Conclusion:**

In conclusion, the chronic exposure to HFD induces sex differences in LS that could be related to pathological mechanisms observed in obesity and to the efficacy of treatment with GLP-1R agonists. Moreover, phentermine, a classic anorectic drug used for short-term weight loss, generates neurobiological adaptations that increase fasting-induced caloric intake, which could be related to the weight regain observed in obese patients.

## 1 Introduction

Obesity has become a serious public health issue, as its prevalence has increased significantly over the past few decades. According to the World Health Organization, the percentage of obese adults rose from 7% to 16% between 1990 and 2022 (WHO, 2024). The primary cause of obesity is an imbalance between caloric intake and energy expenditure, which is influenced by several factors, including sedentary lifestyles, hormonal alterations, and genetic background (Schwartz et al., 2017; Bluher, 2019). Additionally, the high prevalence of obesity is associated with the industrialization of food production, which has promoted the intake of ultra-processed foods rich in macronutrients such as fat and sugar, both highly caloric and palatable (Marti Del Moral et al., 2021).

Several physiological alterations occur in response to obesity and chronic exposure to obesogenic diets, affecting feeding behavior. Some of these central and peripheral alterations include changes in dopamine (DA) neurotransmission (Johnson and Kenny, 2010; Plaza-Briceno et al., 2023; van Galen et al., 2023), leptin and insulin resistance, and dysregulation of the hypothalamic–pituitary–adrenal (HPA) axis (Dallman et al., 2004). Some of these changes are reversed following return to a chow diet and subsequent body weight loss, while others appear to be permanent (Carlin et al., 2013; Altherr et al., 2021).

One physiological system involved in feeding control is the glucagon-like peptide 1 (GLP-1) and its receptor, GLP-1R. GLP-1 is an incretin peptide produced by gut L cells and preproglucagon (PPG) neurons in the nucleus of the solitary tract (NTS) (Muller et al., 2019). In addition, circulating postprandial levels of GLP-1 activate vagal neurons afferent to the central nervous system, which in turn activate the NTS, generating anorexigenic signaling (Krieger, 2020). On the other hand, the activation of intestine-fugal neurons by GLP-1 produces gastrointestinal distension, which decreases food intake at the central level (Zhang et al., 2022). In rodents, PPG neurons are activated by both psychogenic stress and the consumption of large meals, such as those occurring during refeeding (Holt et al., 2019). Activation of these neurons suppresses food intake during refeeding, whereas their inhibition has been demonstrated to increase food intake under the same conditions (Holt et al., 2019; Brierley et al., 2021). PPG neurons send projections to multiple brain regions implicated in the modulation of hedonic aspects of feeding, including the ventral tegmental area (VTA) and nucleus accumbens (NAc) (Alhadeff et al., 2012), and also project to zones involved in the homeostatic control of feeding, such as hypothalamic nuclei (Vrang et al., 2007; Randolph et al., 2024).

Another brain area that receives projections from PPG neurons is the lateral septum (LS) (Rinaman, 2010; Williams et al., 2018), a GABAergic nucleus that expresses the GLP-1R (Graham et al., 2020). The pharmacological activation of GLP-1R in the LS suppresses food intake (Chen et al., 2024). However, the precise physiological source of GLP-1R activation in LS remains unresolved.

The LS participates in the physiological regulation of motivated behaviors, stress responses, homeostasis, and feeding, among other functions (Swanson and Cowan, 1979; Risold and Swanson, 1997; Sheehan et al., 2004). This area also neurochemically regulates the lateral hypothalamus (LH)—commonly referred to as the hunger center (Sweeney and Yang, 2016)—and indirectly activates VTA dopaminergic neurons (Sotomayor et al., 2005; Luo et al., 2011; Vega-Quiroga et al., 2018). In this context, activation of LS neurotensin neurons projecting to the nucleus tuberalis lateralis, located in the posterior hypothalamus, reduces the intake of palatable foods and sweet solutions (Chen et al., 2022). Conversely, a subset of PVN glutamatergic neurons (MC_4_R-positive) that project to and activate the ventral part of the LS also reduce food intake (Xu et al., 2023).

The LS expresses high levels of GLP-1R (Goke et al., 1995; Merchenthaler et al., 1999; Graham et al., 2020), whose activity mediates the effects of alcohol and cocaine (Harasta et al., 2015; Reddy et al., 2016; Allingbjerg et al., 2023; Edvardsson et al., 2025). In addition, GLP-1R–positive neurons in the LS (LS^GLP-1R^) modulate food intake and are pharmacological targets of GLP-1R agonists. In this sense, systemic injection of exendin-4 (Ex4) and liraglutide, two GLP-1R agonists, or the chemogenetic activation of these neurons reduce food intake (Azevedo et al., 2020; Chen et al., 2024). This feeding behavior is associated with an increase in the c-Fos expression and the spontaneous excitatory postsynaptic currents in LS^GLP-1R^ when liraglutide was microinjected into this nucleus (Chen et al., 2024). In this sense, LS Ex4 microinjections reduce food intake, while microinjections of exendin-9, a GLP-1R antagonist, increase food intake and block stress-induced hypophagia (Terrill et al., 2016; Terrill et al., 2018; Terrill et al., 2019).

Interestingly, stressful situations requiring active coping activate LS neurotensin-positive neurons (LS^Nts^) (Azevedo et al., 2020). Specifically, acute exposure to restraint stress produces an increase in c-Fos expression in LS^Nts^, being associated with a reduction in the intake of chow diet (Azevedo et al., 2020). On the other hand, the restraint stress increases the activity of LS^GLP-1R^ and reduces the food intake in male mice fed a control diet (Bales et al., 2022). However, in obese male mice, restraint stress does not increase the activity of hypophagia-inducing LS^GLP-1R^ (Bales et al., 2022). These effects were not observed in female mice, which is concordant with studies showing that the LS is a sexually dimorphic area (Bredewold et al., 2014; VanRyzin et al., 2020).

Recently, the role of the LS in the feeding regulation and the central effects of GLP-1/GLP-1R system activation have emerged as a topic of interest. However, whether chronic exposure to a high-fat diet (HFD) affects this incretinergic system in the LS remains poorly understood. The aim of our work was to investigate whether obesity induces changes in the GLP-1/GLP-1R system and neurotransmitter content in the LS, a relay station that integrates neurotransmitter and neuropeptide signals that regulate feeding. In addition, we studied the effects of post-fasting refeeding behavior and LS GLP-1R content in control and obese rats exposed to dietary and pharmacological treatment with phentermine, a classical sympathomimetic amine anorectic agent. Phentermine has been used for decades in America for the short-term treatment of overweight and obesity in programs that include diet and exercise. However, the adaptive mechanisms that lead to weight regain with this type of drug have not yet been fully elucidated.

## 2 Materials and methods

### 2.1 Reagents

DA, glutamate, and GABA standards, EDTA, and 1-octanesulfonic acid were purchased from Sigma-Aldrich, Inc. (St. Louis, Missouri, USA). Phentermine (Sentis®, ISPCH N°F-19324/22, Laboratorio Chile S.A., Santiago, Chile) was commercially acquired. All other reagents were of analytical and molecular grade.

### 2.2 Animals

One hundred thirty-six males and 138 female Sprague Dawley rats from different litters were used. All animals were housed in a temperature- and humidity-controlled room (22 °C ± 2 °C; 50% ± 5%, respectively) under artificial illumination (12-h light/12-h dark; light on at 08:00 a.m.), with food and water *ad libitum*. All experimental procedures were approved by the Ethics (Act N° BEA154-20; BEA201-24 and CBC62-2022) and Biosafety (Act N° BS002–20) Committees from the Universidad de Valparaíso and the Institutional Animal Experimentation Ethics Board and the Science Council (FONDECYT) of Chile. Efforts were made to minimize the number of rats used and their suffering.

### 2.3 Experimental design

All the animals (n = 274) from the post-natal day (PND) 21 to PND 62, were divided into four experimental groups: Control males (n = 60), high-fat diet (HFD) males (n = 62), control females (n = 62) and HFD females (n = 62). The control groups were fed with chow diet (Prolab® Isopro® RMH 3000, LabDiet, USA), whose composition was 26%, 14% and 60% of kcal from proteins, fats, and carbohydrates, respectively), and the HFD groups were fed with HFD (D12492, Research Diets, Inc., NJ, USA), which composition was 20%, 60% y 20% of kcal from proteins, fats, and carbohydrates, respectively). In addition, to increase the total calorie intake, we used a previously published strategy where the authors incorporated a sucrose solution as drinking water for rats fed with HFD (Rijnsburger et al., 2019). Considering the duration of our protocol (6 weeks), we decided to use a 5% (^w^/_v_) sucrose solution to induce higher caloric intake without producing other metabolic alterations.

#### 2.3.1 Experiment 1

One hundred forty-one animals were used in experiment 1. At PND 62, control (n = 70) and HFD (n = 71) animals were deeply anesthetized with isoflurane (5% in 0.6 L/min air flow) in an induction chamber using an animal anesthesia system (model 510, RWD Life Science Co. Ltd., Shenzhen, P.R. China), and quickly euthanized by decapitation with a guillotine (model 51330, Stoelting^TM^ Co., Wood Dale, IL, USA). After decapitation, the brains were removed and microdissected on ice (at approximately 4 °C degrees) using a brain matrix (model 68711, RWD Life Science, Shenzhen, P.R. China) and micro-punch (model 15076; diameter 2.0 mm, Harris Uni-Core, Ted-Pella Inc., Redding, CA, USA) as was described previously (Venegas et al., 2024). The entire LS was weighed on an analytical balance (model JK-180, Chyo, Japan) and stored at −80 °C for the following analysis: i) GLP-1R protein levels by Westen Blot (Figures 2B,C), ii) GLP-1R expression by RT-qPCR (Figure 2E), iii) GLP-1 levels by ELISA (Figure 2F), and iv) Neurotransmitters content by HPLC coupled electrochemical detection (ED). Specifically, we quantified GABA, dopamine, and glutamate (Figures 3A–D).

#### 2.3.2 Experiment 2

One hundred thirty-three animals were used in experiment 2. At PND 62, control (n = 66) and HFD (n = 67) animals were exposed to a fasting period to measure refeeding behavior at PND 63 and 75. From PND 63 to 74, control and HFD rats had *ad libitum* access to a chow diet and tap water. Control and HFD groups were divided into three subgroups: Saline (1 mL/kg/day, i.p.; n = 38), phentermine (30 mg/kg/day, i.p.; n = 39), and Pair-fed (fed with the same amount of food consumed by phentermine animals, plus saline injection (n = 56) (Figure 3). Each injection was administered for 10 days and after the 2nd refeeding test (PND 75), rats were deeply anesthetized with isoflurane and euthanized by decapitation with guillotine. Their brains were removed, LS microdissected on ice, and stored at −80 °C for the following analysis: i) GLP-1R and D_2_ protein levels by Western blot.

### 2.4 Behavioral experiments: refeeding test

At PND 62 and PND74, the food was removed from the cages 3 hours before the dark cycle (05:00 p.m.), and animals were only given tap water. The next day (PND 63 or 75), from 08:00 until 09:30 a.m., rats were individually housed in special cages with grilled floors and two modules to measure food intake for habituation (BioDAQ unplugged rat cages, New Brunswick, NJ, USA). From 09:30 a.m., one module was filled with control food and another with HFD. After 1 hour (10:30 a.m.), the intake of each kind of food was measured, and the animals were returned to their original cages.

### 2.5 *Ex vivo* analyses

#### 2.5.1 Western blots

LS obtained from experiments 1 and 2 were used to determine GLP-1R protein levels at PND 62 and PND 75. For the protein extraction, tissues were homogenized with RIPA buffer (pH = 8.0, 150 mM NaCl, 50 mM Tris-HCl, 1% (^v^/_v_) Nonidet P40, 0.1% (^w^/_v_) sodium dodecyl sulphate (SDS), 2 mM EDTA, 1.5 mM PMSF, and a protease inhibitor cocktail (Catalog N° G6521, Protease Inhibitor Cocktail 50X, Promega™ Corporation, Madison, WI, USA) using a sonicator (Q55 sonicator, QSonica, Newtown, CT, USA). Total protein concentration was determined by the Bio-Rad Protein Assay (Bio-Rad Laboratories, Inc., Richmond, CA, USA) using a microplate spectrophotometer (Epoch™, BioTek Instruments Inc., Winooski, VT, USA) and protein samples (30 μg) were separated by SDS-PAGE on 8% polyacrylamide gels under denaturing conditions (4% concentrator gel, 10% resolute gel). Proteins were transferred to nitrocellulose membrane (Catalog N° 88018, 0.45 μm pore, Thermo Fisher Scientific Inc., Waltham, MA, USA) at 350 mA for 1.5 h. Non-specific membrane binding sites were blocked with 5% skim milk in T-TBS (0.1% Tween-20, 20 mM TBS, 137 mM NaCl) for GAPDH and BSA 5% in T-TBS was used for GLP-1R, during 1 h at room temperature. Nitrocellulose membranes were incubated with T-TBS overnight at 4 °C and then incubated with the rabbit anti-GLP-1R antibody (Cat N° ab218532, Abcam, Cambridge, MA, USA) (1:1.000, overnight incubation) and rabbit anti-GAPDH antibody (Cat N° ab9485, Abcam, Cambridge, MA, USA) (1:10.000, 1 h incubation). Regard the anti-GLP-1R antibody used in this work, several published papers using the same antibody have reported in peripheral tissues bands for GLP-1R at 46 (Shi et al., 2023) and 53 (Kajitani et al., 2023) kDa. In addition, this antibody was validated using a GLP-1R knockout mouse (Kajitani et al., 2023) and others brain areas a band of approximately 80 kDa was detected (Greenwood et al., 2023).

Finally, the antibody complexes were detected using a goat anti-rabbit IgG Fc conjugated with HRP (Catalog N° ab97200, Abcam, Cambridge, UK). For chemiluminescent detection, we used an EZ-ECL kit (Catalog N° 20–500–500, Biological Industries Ltd, Beit Haemek, Israel), and the images of the membranes were obtained using Omega Lum™ G (Gel Company, San Francisco, CA, USA) and the images were analyzed using ImageJ™ software (http://rsbweb.nih.gov/ij/).

#### 2.5.2 RT-qPCR

Real-time RT-qPCR was used to determine changes in the mRNA encoding GLP-1R in LS at PND 62. Total RNA was extracted using the E.Z.N.A. Total RNA Kit I (Catalog N° R6834-02, Omega Biotek, Inc., Norcross, GA, USA) according to the manufacturer’s instructions. RNA was quantified using a microplate spectrophotometer (Epoch™, BioTek Instruments Inc., Winooski, VT, USA), and RNA integrity was assessed through agarose gel electrophoresis. Total RNA from each sample was reverse transcribed with PrimeScript RT reagent Kit (Catalog N° RR047A, TaKaRa, Bio Inc., CA, USA), according to the manufacturer’s instructions. Real-time RT-PCR was performed using TaqMan assays for GLP-1R (Catalog N° Rn00562406_m1, FAM dye-labeled, Life Technologies Corporation, CA, USA) and 18s (Catalog N° Cat: Rn03928990_g1, VIC dye-labeled, Life Technologies Corporation, CA, USA) genes. The cycle conditions were 50 °C for 2 min, 95 °C for 2 min, 40 cycles for 95 °C for 3 s and 60 °C for 30 s. Results were expressed as fold change by the 2^−ΔΔCT^ (Livak and Schmittgen, 2001).

#### 2.5.3 ELISA

Because the GLP-1 peptide is small, making it difficult to detect by Western blot, we quantified it using an ELISA. LS GLP-1 peptide content was measured following the manufacturer’s instructions described in the ELISA Kit (Catalog N° BMS2194, Invitrogen, Thermo Fisher Scientific Inc., Waltham, MA, USA). LS tissue was collected in the absence of DPP-4 inhibitors and rinsed with 1X PBS and homogenized in 500 µL of 1X PBS using a sonicator (Q55 sonicator, QSonica, Newtown, CT, USA). The homogenates were centrifuged for 5 min at 5,000 x g, 4 °C. The supernatant was removed and stored at −20 °C for further analysis.

#### 2.5.4 Neurotransmitter tissue content

LS was weighed on an analytical balance and homogenized in 0.4 mL of 0.2M perchloric acid using a sonicator (Q55 sonicator, QSonica, Newtown, CT, USA) in ice. The homogenate was centrifuged to 12,000 g for 15 min at 4 °C (model Z233MK-2, Hermle Labor Technik GmbH, Wehingen, Germany), and the supernatant was filtered using HPLC syringe filters (model EW-32816-26; Cole-Parmer, Vernon Hills, IL, USA).

##### 2.5.4.1 DA quantification

Ten microliters of final clear supernatant were injected into HPLC-ED with the following configuration: Isocratic pump (model PU-2080 Plus, Jasco Co. Ltd., Tokyo, Japan), C18-column (model Kromasil 100-3.5-C18, AkzoNobel, Bohus, Sweden), and electrochemical detector (model LC-4C, Bioanalytical System Inc., West Lafayette, IN, USA) set at 0.700 V (oxidation potential), 0.5 nA (sensitivity), and 0.03 Hz (electrical noise). The composition of the mobile phase was 0.1 M NaH_2_PO_4_, 1.5 mM 1-octanesulfonic acid, 1.28 mM EDTA, 2.0% (^v^/_v_) tetrahydrofuran, and 4.5% (^v^/_v_) CH_3_CN (pH 3.6). It was pumped at a flow rate of 0.15 mL/min, and the retention time for DA was 11.7 min. The quantification was performed using a calibration curve for DA (Program ChromPass, Jasco Co. Ltd., Tokyo, Japan). The concentration was expressed as pg per mg of wet tissue.

##### 2.5.4.2 Glutamate and GABA quantification

Twenty microliters of final clear supernatant were mixed with 4 μL of borate buffer (pH 10.8), and 4 μL of fluorogenic reagent (20 mg of orthophthaldehyde and 10 μL of β-mercaptoethanol in 5 mL of ethanol absolute). The mixture was shaken for 90 s to complete the derivatization and injected into HPLC coupled to fluorometric detection (FD) with the following configuration: Isocratic pump (model PU-4180, Jasco Co. Ltd., Tokyo, Japan), C18-column (model Kromasil 100-3.5-C18, AkzoNobel, Bohus, Sweden), and fluorescence detector (model FP-4025, Jasco Co. Ltd., Tokyo, Japan). The composition of the mobile phase was 0.1 M NaH_2_PO_4_ and 24.0% (^v^/_v_) CH_3_CN (pH 5.7). It was pumped at a flow rate of 0.8 mL/min, and the retention times for Glutamate and GABA were 1.5 and 13.0 min, respectively. The quantification was performed using a calibration curve for Glutamate and GABA (Program ChromNAV 2.0, Jasco Co, Ltd, Tokyo, Japan). The concentration was expressed as ng per mg of wet tissue.

### 2.6 Statistical analysis

**FIGURE 1:**
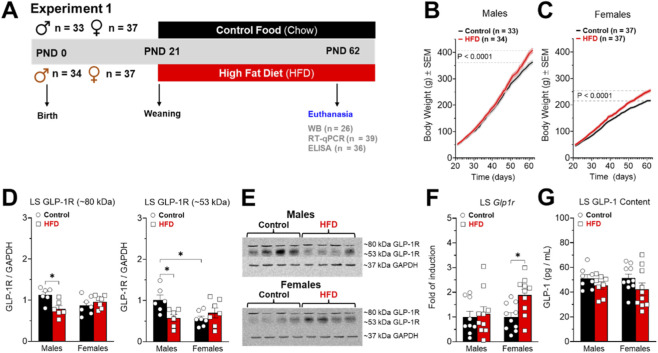
**(A)** Timeline experiment 1. Effects of high-fat diet (HFD) on the body weight of male (**(B)**, n = 67) and female (**(C)**, n = 74) rats, and on the lateral septum (LS) GLP-1/GLP-1R system **(D–G)**. **(D)** GLP-1R protein levels in the LS of control (male = 6 and female = 7) and HFD (male = 6 and female = 7) rats. The data (mean ± SEM) are arbitrary units of GLP-1R immunoreactivity normalized to GAPDH immunoreactivity. **(E)** Representative bands at ∼80 and ∼53 kDa for GLP-1R and ∼37 kDa for GAPDH. **(F)** *Glp1r* gene expression in the LS of control (male = 9 and female = 10) and HFD (male = 10 and female = 10) rats. All data (mean ± SEM) have been normalized for levels of 18S mRNA expression within the same sample. **(G)** LS GLP-1 peptide levels of control (male = 8 and female = 10) and HFD (male = 8 and female = 10) rats. Results are expressed as mean ± SEM. Mann-Whitney test was used to analyze panels **(B)** and **(C)**. Two-way ANOVA Tukey´s multiple comparisons test was used to analyze panels **(D,F,G)**. *P < 0.05.

**FIGURE 2:**
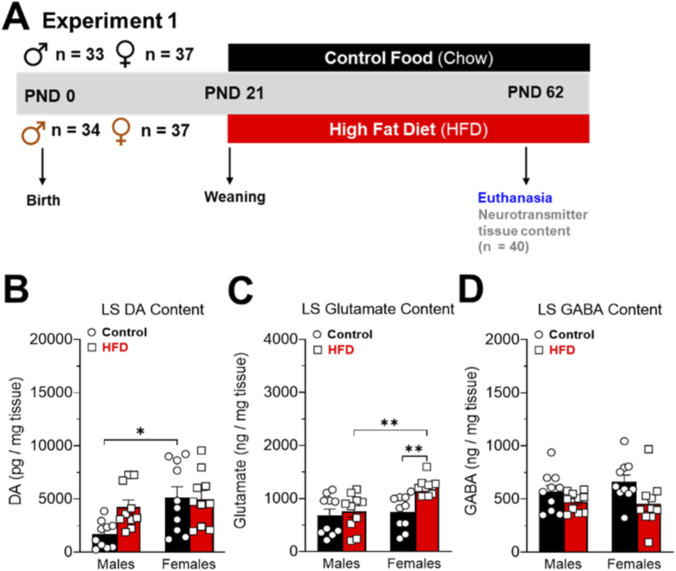
**(A)** Timeline experiment 1. Effects of high-fat diet (HFD) on the dopamine **(B)**, glutamate **(C)**, and GABA **(D)** tissue levels in the LS of control (male = 10 and female = 10) and HFD (male = 10 and female = 10) rats. The data (mean ± SEM) are expressed in pg/mg of tissue. Two-way ANOVA Tukey´s multiple comparisons test was used to analyze panels **(B–D)**. *P < 0.05, **P < 0.01.

**FIGURE 3:**
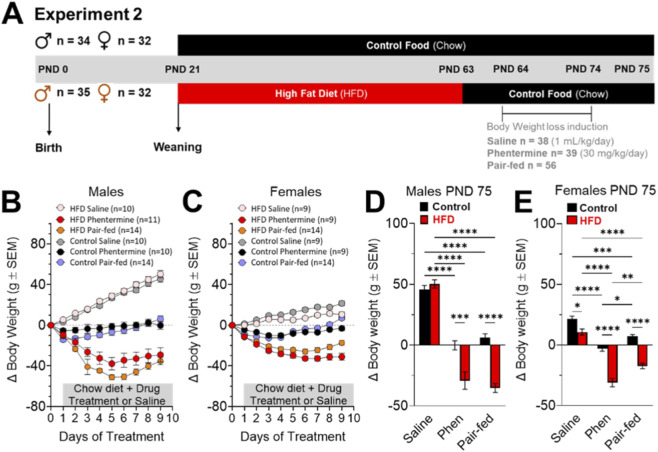
**(A)** Timeline experiment 2. Effects of dietary and pharmacological treatments on body weight of control (male = 34 and female = 32) and HFD (male = 35 and female = 32) rats. **(B,D)** Show the weight gain of control (saline = 10, Phentermine = 10 and Pair-fed = 14) and HFD (saline = 10, Phentermine = 11 and Pair-fed = 14) male rats. **(C,E)** Show the weight gain of control (saline = 9, Phentermine = 9 and Pair-fed = 14) and HFD (saline = 9, Phentermine = 9 and Pair-fed = 14) female rats. The data (mean ± SEM) are expressed in g of body weight. Two-way ANOVA Tukey´s multiple comparisons test was used to analyze panels **(D,E)**. *P < 0.05, **P < 0.01, ***P < 0.001, ****P < 0.0001.

**FIGURE 4:**
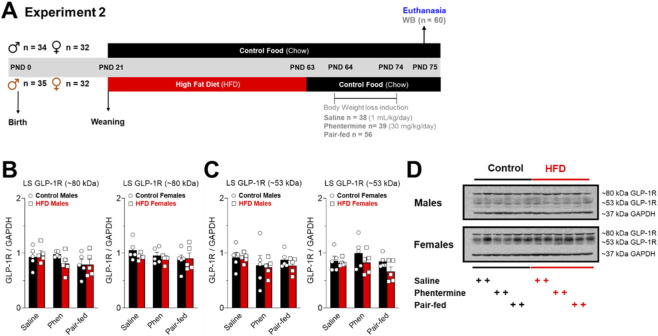
**(A)** Timeline experiment 2. Effects of dietary and pharmacological treatments on GLP-1 receptor (GLP-1R) protein levels in the LS of control (male = 15 and female = 15) and HFD (male = 15 and female = 15) rats. **(B,C)** Show the bands at ∼80 kDa **(B)** and ∼53 kDa **(C)** for GLP-1R in the LS of control (saline = 10, Phentermine = 10 and Pair-fed = 10) and HFD (saline = 10, Phentermine = 10 and Pair-fed = 10) male and female rats. **(D)** Representative bands at ∼80 and ∼53 kDa for GLP-1R and ∼37 kDa for GAPDH. The data (mean ± SEM) are arbitrary units of GLP-1R immunoreactivity normalized to GAPDH immunoreactivity. Two-way ANOVA Tukey´s multiple comparisons test was used to analyze panels **(B,C)**.

**FIGURE 5:**
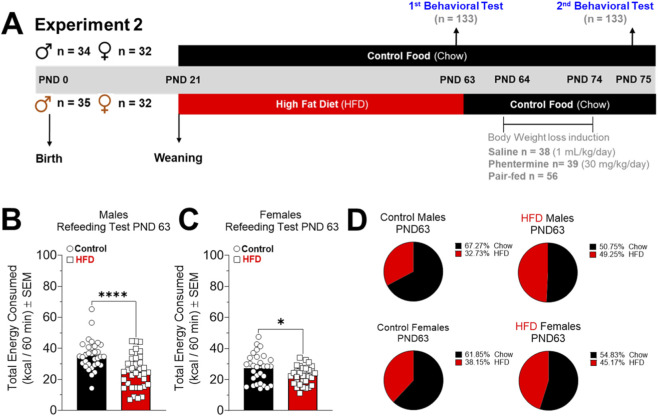
**(A)** Timeline experiment 2. Effects of high-fat diet (HFD) on food intake in a refeeding test in control (male = 34 and female = 32) and HFD (male = 35 and female = 32) rats at post-natal day (PND) 63 and PND 75. **(B,C)** Show the total kcal intake after 6 weeks of exposure to HFD in male and female rats, respectively. **(D)** Show the percentage of chow intake versus HFD intake. Results (mean ± SEM) are expressed in kcal/60 min, and a t-test was used to analyze panels **(B,C)**. *P < 0.05, ****P < 0.0001.

**FIGURE 6:**
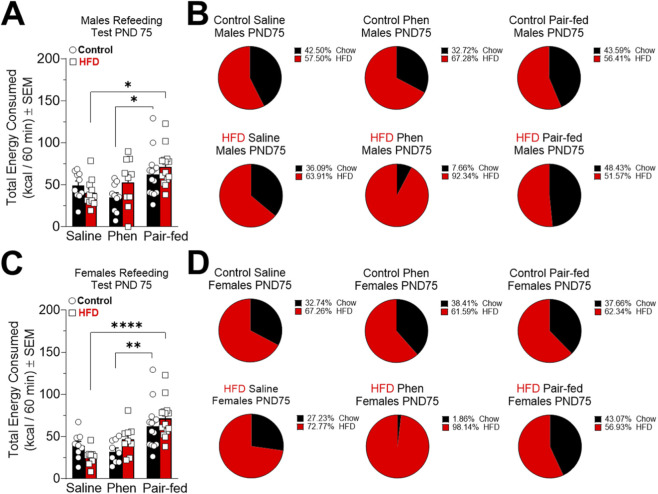
Effects of high-fat diet (HFD) and subsequent pharmacological and dietary treatments on food intake in a refeeding test in control (male = 34 and female = 32) and HFD (male = 35 and female = 32) rats at post-natal day (PND) 75. **(A,C)** Show total kcal intake after 10 days of dietary and pharmacological treatments in control male (saline = 10, Phentermine = 10 and Pair-fed = 14), control female (saline = 9, Phentermine = 9 and Pair-fed = 14), HFD male (saline = 10, Phentermine = 11 and Pair-fed = 14) and HFD female (saline = 9, Phentermine = 9 and Pair-fed = 14) rats. **(B,D)** Shows the percentage of chow intake versus HFD intake. The data (mean ± SEM) are expressed in kcal/60 min. Two-way ANOVA Tukey´s multiple comparisons test was used to analyze panels **(A,C)**. *P < 0.05, **P < 0.01, ****P < 0.0001.

Results were expressed as mean ± SEM. Figures 1D,F,G, 2–4, 6A,C were analyzed using 2-way ANOVA with Tukey’s multiple comparisons. Figures 1B,C, 5B,C were analyzed using Mann–Whitney test. The statistical analyses were carried out with GraphPad Prism version 10.5.0 (GraphPad Software, San Diego, CA, USA), and P < 0.05.

## 3 Results

### 3.1 Exposure to an obesogenic diet for 6 weeks produces sex differences in the LS GLP-1/GLP-1R system in rats

To evaluate whether exposure to obesogenic diets alters the GLP-1 system in the LS, we exposed male and female rats to an HFD combined with a 5% w/v sucrose solution for 6 weeks. After these 6 weeks, at PND 62, we measured levels of gonadal and retroperitoneal fat to obtain a more comprehensive assessment of diet-induced adiposity. We also performed ELISA, RT-qPCR, and Western blot to assess GLP-1 peptide levels, *Glp1r* gene expression, and GLP-1R protein levels in the LS, respectively (Figure 1A). Males and females exposed to the HFD showed increased body weight at PND 62 and increased the accumulation of retroperitoneal and gonadal fats.

Although Western blot is a semi-quantitative technique that generally provides a relative estimation of protein abundance, the specificity and sensitivity of this technique allow the comparison of protein expression levels between different samples, including post-translational modifications. In this context, we observed a reduction in GLP-1R protein levels in the LS of obese male rats (Figure 1D, band at 80 kDa and band at 53 kDa). The band of 80 kDa for GLP-1R undergoes glycosylation, a post-translational modification that delays its migration on protein gels (Greenwood et al., 2023) and both bands for GLP-1R were detected in LS (Figure 1E) and nucleus accumbens (Supplementary Figure 2). Tibial muscle and pancreas were used as negative and positive controls, respectively, showing two bands at 46 and 53 kDa (Supplementary Figure 2). Both bands have been previously detected in previous publications where the same antibody detected the 53 kDa band in lung tissue (Kajitani et al., 2023) and the 46 kDa band in kidney tissue (Shi et al., 2023). Additionally, the antibody was validated using knockout mice for the GLP-1R receptor (Kajitani et al., 2023).

Regarding gene expression, we observed that HFD exposure increased *Glp1r* expression in the LS only in obese female rats and it has not effect in males. The discrepancy between the results obtained by WB and PCR could be attributed to post-translational glycosylation, which may be altered in males but not in females. Other mechanisms could include compensatory changes in protein synthesis or degradation. Finally, HFD exposure did not affect GLP-1 peptide levels in the LS of male or female rats, indicating that in our obesity model, the primary HFD-induced changes occur at the level of GLP-1R protein expression in a sex-dependent manner.

### 3.2 Exposure to an obesogenic diet for 6 weeks produces sex differences in LS glutamate content in rats

Next, we aimed to evaluate whether exposure to the obesogenic diet also alters neurotransmitter content in the LS. To this end, we analyzed tissue samples using HPLC-coupled to electrochemical detection to measure DA and HPLC-coupled to fluorescent detection to measure amino acid neurotransmitters. Six weeks of HFD exposure did not alter DA content in the LS of male or female rats. Additionally, LS DA content was higher in control female rats compared to control males, indicating a baseline sex difference as it was described in other nuclei (Sotomayor-Zarate et al., 2014; Yoest et al., 2019). In contrast, glutamate content was increased in the LS of HFD female rats but not in males. Also, HFD females show higher glutamate content than HFD males (P < 0.01). The diet did not affect LS GABA content.

### 3.3 Dietary and pharmacological treatment for weight loss reverses the reduction of LS GLP-1R levels in HFD male rats

Next, we aimed to evaluate whether the diet-induced alterations in the GLP-1 system in the LS persist after weight loss. After 6 weeks of exposure to an obesogenic diet, animals were treated for 10 days with a control diet and phentermine (a sympathomimetic drug with an anorectic effect) to reduce body weight (Figure 3A). During this period, all rats were fed with standard chow and divided into three groups: i) The saline group received a daily intraperitoneal (i.p.) injection of saline solution (1 mL/kg), ii) the phentermine group received a daily i.p. injection of phentermine (30 mg/kg, i.p.), and iii) the pair-fed group was provided the same amount of food consumed by the phentermine-treated animals, along with vehicle injections.

Dietary and pharmacological treatment resulted in weight loss in animals exposed to HFD for 6 weeks, while saline injections-maintained weight gain curves (Figures 3B,C). After 10 days of treatment, phentermine and pair-fed groups showing significant reduction of the body weight in male and female rats (Figures 3D,E). In males, phentermine and pair-fed groups showed lower retroperitoneal fat levels compared with the saline-treated group (Supplementary Figure 3A), while the pair-fed group only showed a reduction in gonadal fat pad compared with the saline group (Supplementary Figure 3B). In females, phentermine reduced retroperitoneal and gonadal fat pads (Supplementary Figures 3C,D).

Twenty-four hours after dietary and pharmacological treatment, the animals were euthanized, and GLP-1R protein levels were measured in LS (Figure 4A). Interestingly, dietary treatment by itself reversed the reduction in LS GLP-1R levels of male rats exposed to HFD for 6 weeks (Figure 4B). Additionally, no changes in receptor protein levels were observed in the LS female rats of saline, phentermine and pair-fed groups (Figure 4C).

### 3.4 Exposure to an obesogenic diet for 6 weeks affects post-fasting refeeding behavior

We evaluated the effects of chronic exposure to HFD on post-fasting refeeding behavior before starting (PND 63) and 24 h after dietary and pharmacological treatment (PND 75) (Figure 5A). After 16.5 h of fasting (see material and method, Section 2.4), the type of food consumed by the animals was measured. At PND 63, HFD male and female rats showed significantly lower total energy intake compared to control animals (Figures 5B,C). Additionally, control animals consumed more control food than HFD, while HFD rats of both sexes had a similar consumption of control and HFD food in the post-fasting refeeding test (Figure 5D; Supplementary Figures 4B,C).

When we evaluated whether these changes in re-feeding behavior were maintained or reversed following weight loss, we observed no differences in total energy intake between control and HFD rats that received saline injections along with a control diet (Figures 6A,B). Pair-fed rats, regardless of sex or dietary group, consumed more total energy and experienced greater weight loss during fasting compared to saline and phentermine-treated animals (Figures 6A,C).

Interestingly, at PND 75, the HFD rats of both sexes treated with phentermine showed markedly higher HFD intake in the post-fasting refeeding test, despite having lost weight and having been fed a control diet for the previous 10 days. This effect was not observed in the control groups or in the saline and pair-fed HFD groups (Figures 6B,D; Supplementary Figures 4D,E).

## 4 Discussion

This study aimed to determine whether diet-induced obesity leads to alterations in the GLP-1/GLP-1R system and within the LS, and whether such changes are reversible through weight-loss interventions. Interestingly, our findings indicate that exposure to HFD induces sex-specific alterations in the LS GLP-1 system, increasing *Glp1r* expression in females and decreasing GLP-1R protein levels in males. In this context, GLP-1/GLP-1R system exhibits some sex differences, showing that females treated with antidiabetic drugs such as liraglutide and semaglutide, both GLP-1R agonists, lose more weight but also experience more adverse events than males (Rentzeperi et al., 2022). Moreover, circulating GLP-1 levels tend to be higher in women than in men (Adam and Westerterp-Plantenga, 2005; Faerch et al., 2015). In animal studies, females were more sensitive to the effects of GLP-1R agonists on food intake and body weight reduction than males (Liang et al., 2018). Hence, the reduction in LS GLP-1R levels in obese male rats could be related to a lower efficacy of GLP-1R agonists in these animals. In females, 17β-estradiol (E2), the main estrogenic hormone, plays a regulatory role in feeding, showing an anorexigenic effect (appetite-suppressing) mediated by the activation of estrogen receptor alpha (Erα) (Santollo et al., 2007; Thammacharoen et al., 2008). In addition, the exposure to HFD induces metabolic syndrome only in males and ovariectomized female animals (Rodriguez-Rodriguez et al., 2021), showing a protective effect of E2 in HFD female rats (Riant et al., 2009). Finally, ERα signaling seems to be involved in the effect of Ex4, GLP-1R agonist, on food reward reduction in rats, where females are more sensitive to this effect (Richard et al., 2016). Although, LS has shown sex differences in behaviors such as inhibiting lordosis in females (Tsukahara and Yamanouchi, 2001) and inducing anxious behaviors in males (Hasunuma et al., 2024), the results of this work show for the first time that chronic exposure to HFD produces a clear sex difference in the LS GLP-1/GLP-1R system that could affect the neurobiological mechanisms that regulate feeding and the therapeutic efficacy of GLP-1 agonists to reduce food intake, possibly requiring a dose adjustment for this type of drug according to sex.

The exposure to HFD induces numerous metabolic and physiological alterations, including changes at the central level in systems involved in food intake regulation, such as dopamine neurotransmission and ghrelin/leptin signaling. These changes promote feeding behaviors that impede weight loss (Berthoud et al., 2011; Perez-Leighton et al., 2024). Metabolic dysregulation associated with obesity has been increasingly linked to impairments in gut–brain communication pathways. In particular, obesity has been associated with a reduced responsiveness to anorexigenic gut hormones that normally contribute to regulating appetite and energy balance (Gruber et al., 2025). Regard to GLP-1 system, obese patients present reduced fasting and postprandial plasma levels of GLP-1 (Manell et al., 2016; Jones et al., 2021). Consistent with this notion, several studies have reported an attenuated postprandial GLP-1 response in individuals with obesity (Lean and Malkova, 2016). In addition, emerging evidence suggests that obesity-associated alterations in the gut microbiota may contribute to impaired GLP-1 signaling, potentially promoting a state of GLP-1 resistance (Zeng et al., 2024). Although circulating GLP-1 levels were not measured in the present study, future investigations evaluating both systemic GLP-1 levels and the functional response to gut-derived GLP-1 signaling would provide valuable insight into the mechanisms underlying gut–brain maladaptation in our obesity model.

At the central level, exposure to an obesogenic diet decreases GLP-1R levels in the NTS and hypothalamus (Mul et al., 2013; Mella et al., 2017), and impairs the anorectic effects of GLP-1R activation (Primeaux et al., 2010; Williams et al., 2011; Duca et al., 2013; Mul et al., 2013). The LS participates in the regulation of feeding, receives projections from NTS GLP-1 neurons, and widely expresses GLP-1R; however, there is limited evidence regarding obesity-induced alterations in the LS GLP-1/GLP-1R system. Specifically, Bales et al. demonstrated that chronic exposure to HFD affects stress-induced hypophagia associated with the loss of activation of LS neurons that express GLP-1R (Bales et al., 2022). In this context, our results did not show changes in the LS GLP-1 peptide levels. However, we observed reduced LS GLP-1R levels in HFD male rats. These findings are consistent with the results reported by Bales et al. (2022) and support the hypothesis that the LS GLP-1/GLP-1R system is altered in obesity, making it essential to investigate the mechanisms by which exposure to an obesogenic diet induces these changes. Potential mechanisms may include hormonal alterations, inflammatory factors, or metabolic signaling pathways (Vagena et al., 2019; Wallace and Fordahl, 2022; Plaza-Briceno et al., 2023). On the other hand, new studies are needed to investigate the effects of obesogenic diets on the LS GLP-1/GLP-1R system and its relationship with feeding behavior, since it has recently been shown that the projections of LS GLP-1 positive neurons to the LH are involved in regulating the feeding process (Lu et al., 2024).

Another interesting result related to sex differences was the increase in LS glutamate levels observed in HFD female rats. The exposure to HFD is known to alter neurotransmitter levels in both plasma and the brain (Kim et al., 2013; Labban et al., 2020). In this case, the specific changes in LS neurotransmitter content induced by the HFD may be related not only to alterations in feeding behavior but also to processes such as stress, anxiety, and fear, given the extensive network of connections involving the LS (Rizzi-Wise and Wang, 2021).

In addition, we also found sex differences in LS DA content between, showing higher DA levels in female than male control rats. This dimorphism between sexes may reflect differences in post-transcriptional regulation, protein turnover, or distinct inflammatory responses, which are known to be different between males and females (Braga Tibaes et al., 2024). For example, females are more susceptible to the central inflammatory changes associated with excessive fat and sugar intake, increasing levels of TNFα, IL-1β, IL-6, and IFNγ (Church et al., 2022). These changes in cytokine profile could alter the expression of genes related to food intake (Senaris et al., 2011; Malynn et al., 2013) and have been related to alterations in the brain neurotransmitter pool (Labban et al., 2020).

To determine whether obesity also affects re-feeding behavior, we conducted a re-feeding intake test at PND63, in which males and females exposed to the HFD consumed more total calories than control animals. Other studies have reported that obese animals consume less food during refeeding compared to control animals, and this effect has been linked to alterations in the ghrelin system (Perreault et al., 2004).

Another difference observed between control and HFD animals was the type of food consumed during the refeeding test. At PND 63, control rats consumed more control food, whereas HFD animals consumed similar amounts of both food types. However, at PND 75, control rats injected with saline consumed comparable amounts of control food and HFD. An important consideration when interpreting the refeeding paradigm is that animals were not previously exposed to both diets before the test. Consequently, responses during the refeeding session may reflect not only metabolic drive but also the influence of food familiarity, novelty, or inherent palatability differences between diets. At PND63, control animals may exhibit neophobic behavior upon first-time exposure to HFD. In this context, it has been reported that neophobia can reduce food intake as a protective mechanism against potential illness (Greiner and Petrovich, 2020). Therefore, the intake patterns observed in this paradigm likely represent the combined effects of prior dietary history and acute diet choice following fasting. Importantly, all experimental groups were tested under identical conditions, allowing meaningful comparisons of relative intake between groups despite this limitation. Future studies incorporating prior habituation to both diets would help disentangle the respective contributions of metabolic state, food novelty, and learned diet preference to the observed feeding responses.

There are various strategies to treat obesity and overweight, including lifestyle changes such as diet and exercise (Soini et al., 2016; Cleo et al., 2019), as well as pharmacological options, including drugs with anorectic effects such as phentermine, a sympathomimetic amine used for weight reduction in patients with obesity (Mordes et al., 2015; Singh et al., 2022). However, it remains unclear whether the physiological alterations induced by obesity persist after weight loss. In our work, control and HFD animals were exposed to a weight loss treatment based on phentermine administration combined with a switch to a control diet. In the present study, phentermine was used as a pharmacological tool to induce weight loss through mechanisms that do not directly target the GLP-1/GLP-1R signaling pathway. This approach allowed us to evaluate whether the diet-induced alterations in the GLP-1 system could be reversed by weight loss. Notably, our results indicate that switching animals from HFD to the control diet was sufficient to restore LS GLP-1R levels, even in the absence of marked body weight loss in the saline-treated group. These findings suggest that dietary composition, rather than body weight reduction *per se* or a drug-specific effect of phentermine, may play a role in the recovery of GLP-1R levels. Together, these observations highlight the potential contribution of dietary factors to the regulation of central GLP-1 signaling under conditions of diet-induced obesity.

When caloric intake was experimentally matched through pair-feeding, weight loss in males was comparable between groups. Although phentermine is a sympathomimetic agent whose predominant anti-obesity effect is mediated through central catecholaminergic suppression of appetite, previous studies have also suggested that it may increase energy expenditure (Arch, 1981). In the present study, the lack of additional weight loss in phentermine-treated males relative to pair-fed animals suggests that any potential increase in energy expenditure may have been modest and insufficient to produce further weight reduction under conditions of matched caloric intake. Alternatively, compensatory metabolic adaptations during the intervention could have attenuated potential drug-induced increases in energy expenditure, resulting in similar weight loss between groups.

In HFD animals, the treatment with phentermine plus a control diet increased HFD consumption in the refeeding test. However, this effect was not observed in pair-fed animals despite having a similar weight loss to the obese animals treated with phentermine. In this context, the macronutrient selection following a fasting period has been linked to the activation of DA neurons (Mazzone et al., 2020; Cui et al., 2023), and phentermine acts as a DA releaser (Balcioglu and Wurtman, 1998; Baumann et al., 2000). Therefore, this change in macronutrient selection or preference induced by amphetamine-derived drugs could be part of the neurobiological mechanisms that lead to the rebound effect after weight loss in response to stressors such as prolonged fasting.

In summary, chronic exposure to HFD induces sex-dependent alterations in the LS that may underlie obesity-related pathophysiology and modulate GLP-1R agonist efficacy, while phentermine promotes neurobiological adaptations that enhance fasting-induced caloric intake, potentially contributing to weight regain and to maintain the obesity condition.

## Data Availability

The original contributions presented in the study are included in the article/Supplementary Material, further inquiries can be directed to the corresponding author.
