# Supplementary material for: Diet-induced obesity impairs refeeding responses and downregulates lateral septal GLP-1R in male rats: an effect reversed by weight-loss treatment
Source: Front Pharmacol. 2026 Apr 29;17:1801283. doi: 10.3389/fphar.2026.1801283 (PMC13167487; doi:10.3389/fphar.2026.1801283)
Supplement: Supplementary file 1 [file Supplementaryfile1.docx]

**Supplementary Figure 1**

**
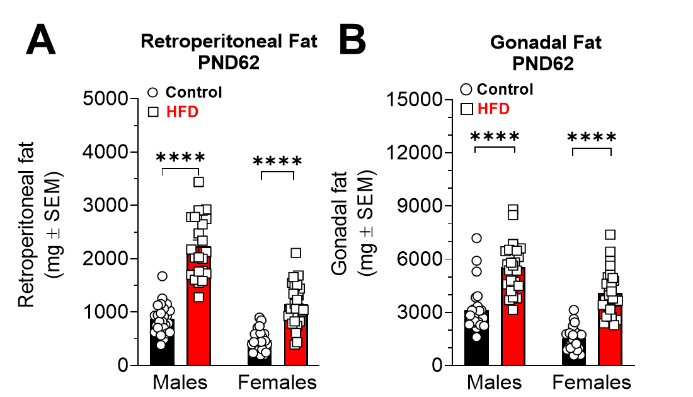
**

**Supplementary Figure 1.** Effect of high-fat diet (HFD) on retroperitoneal (A) and gonadal (B) fat tissues at post-natal day (PND) 62 of control (male = 33 and female = 37) and HFD (male = 34 and female = 37) rats. The data (mean ± SEM) are expressed in g of fat tissue. Two-way ANOVA Tukey´s multiple comparisons test was used to analyze panels (A) and (B). ****P < 0.0001.

**Supplementary Figure 2**

**
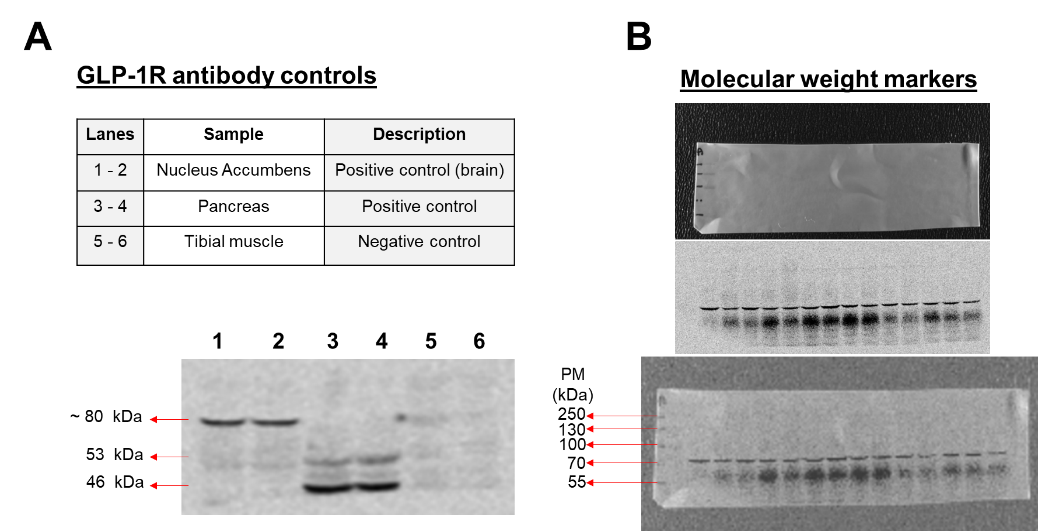
**

**Supplementary Figure 2.** GLP-1 antibody controls. (A) Pancreatic and nucleus accumbens tissues were used as positive controls, while tibial muscle tissue was used as a negative control. We used a band at ~80 kDa observed in the nucleus accumbens and lateral septum for analysis. (B) Antibody signal and molecular weight ladder.

**Supplementary Figure 3**

**
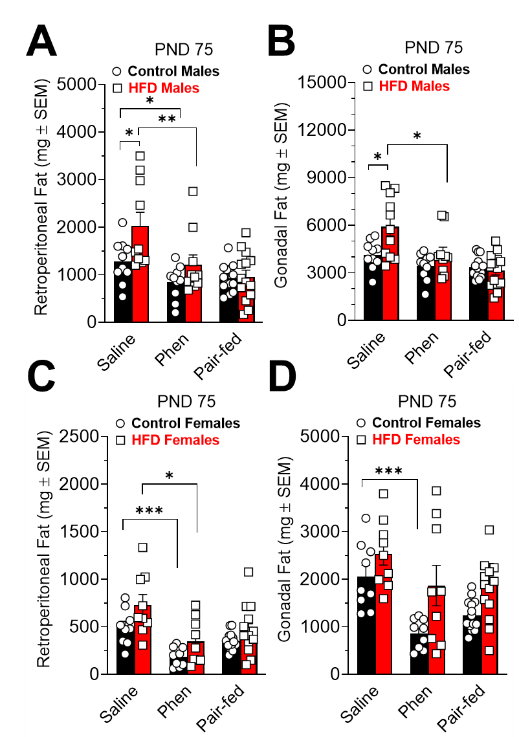
**

**Supplementary Figure 3.** Effect of high-fat diet (HFD) on retroperitoneal (A and C) and gonadal (B and D) fat tissues at post-natal day (PND) 75 of control male (saline = 10, Phentermine = 10 and Pair-fed = 14), control female (saline = 9, Phentermine = 9 and Pair-fed = 14), HFD male (saline = 10, Phentermine = 11 and Pair-fed = 14) and HFD female (saline = 9, Phentermine = 9 and Pair-fed = 14) rats. The data (mean ± SEM) are expressed in g of fat tissue. Two-way ANOVA Tukey´s multiple comparisons test was used to analyze panels (A, B, C and D). *P < 0.05, **P < 0.01, ***P < 0.001, ****P < 0.0001.

**Supplementary Figure 4**

**
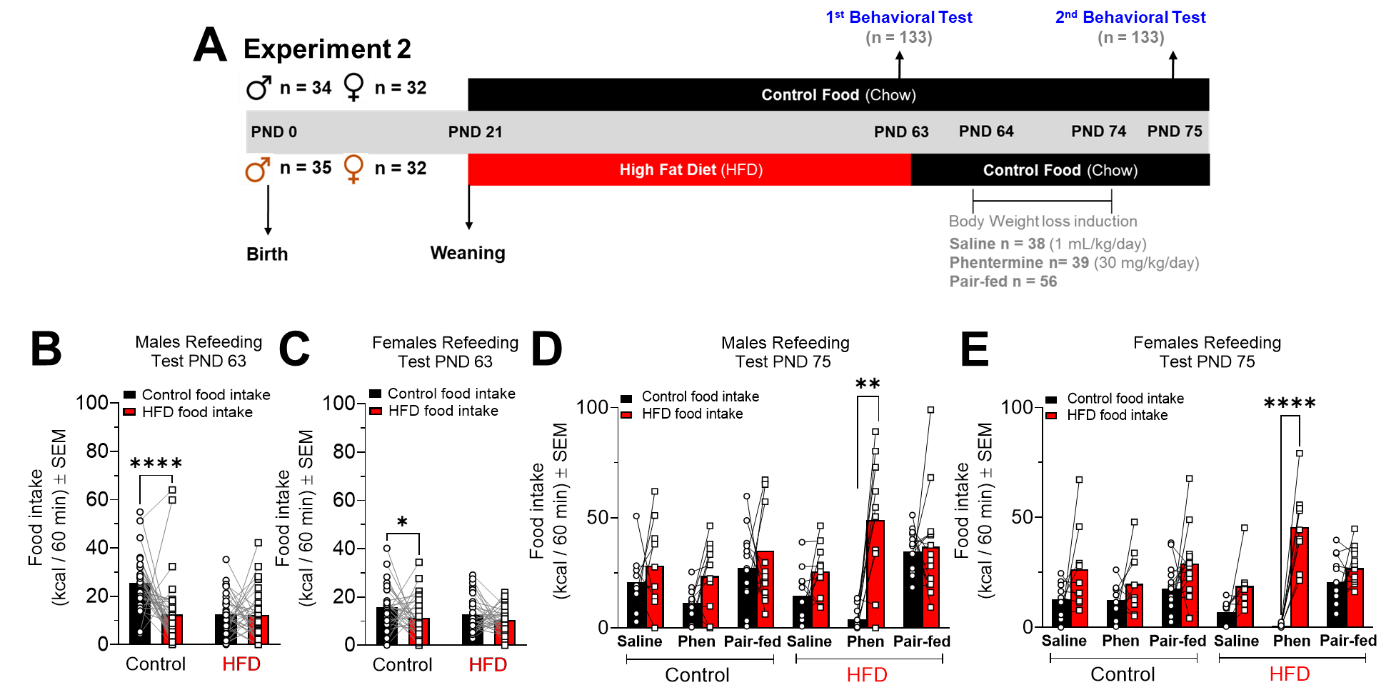
**

**Supplementary Figure 4.** (A) Timeline experiment 2. Effects of high-fat diet (HFD) on food intake in a refeeding test in control (male = 34 and female = 32) and HFD (male = 35 and female = 32) rats at post-natal day (PND) 63 and PND 75. (B) and (C) show food intake after 6 weeks of exposure to HFD in male and female rats, respectively. (D) and (E) show food intake after 10 days of dietary and pharmacological treatments in control male (saline = 10, Phentermine = 10 and Pair-fed = 14), control female (saline = 9, Phentermine = 9 and Pair-fed = 14), HFD male (saline = 10, Phentermine = 11 and Pair-fed = 14) and HFD female (saline = 9, Phentermine = 9 and Pair-fed = 14) rats. The data (mean ± SEM) are expressed in kcal/60 min. Two-way ANOVA Tukey´s multiple comparisons test was used to analyze panels (D) and (E). *P < 0.05, **P < 0.01, ****P < 0.0001.

**Supplementary Table 1.** Statistical information per figure.

| **Figure** | **Parameter** | **Statistical test** | **P value** |
| --- | --- | --- | --- |
| 1B | Body weight (males) | Mann–Whitney test. | P < 0.0001 |
| 1C | Body weight (females) | Mann–Whitney test. | P < 0.0001 |
| 1D | LS GLP-1R protein levels (band at 80 kDa) | 2-way ANOVA with Tukey’s multiple comparisons | Interaction [F_(1,22)_ = 8.367; P = 0.0084] |
|  |  |  | Sex [F_(1,22)_ = 0.3073; P = 0.5850] |
|  |  |  | Diet [F_(1,22)_ = 2.852; P = 0.1054] |
|  | LS GLP-1R protein levels (band at 53 kDa) | 2-way ANOVA with Tukey’s multiple comparisons | Interaction [F_(1,22)_ = 7.967; P = 0.0099] |
|  |  |  | Sex [F_(1,22)_ = 2.636; P = 0.1187] |
|  |  |  | Diet [F_(1,22)_ = 2.2026; P = 0.1686] |
| 1F | LS *Glp1r*expression | 2-way ANOVA with Tukey’s multiple comparisons | Interaction [F_(1,35)_ = 2.583; P = 0.1170] |
|  |  |  | Sex [F_(1,35)_ = 2.600; P = 0.1159] |
|  |  |  | Diet [F_(1,35)_ = 4.669; P = 0.0376] |
| 1G | LS GLP-1 content | 2-way ANOVA with Tukey’s multiple comparisons | Interaction [F_(1,32)_ = 0.1666; P = 0.6859] |
|  |  |  | Sex [F_(1,32)_ = 0.1304; P = 0.7204] |
|  |  |  | Diet [F_(1,32)_ = 3.640; P = 0.0654] |
| 2B | LS dopamine content | 2-way ANOVA with Tukey’s multiple comparisons | Interaction [F_(1,36)_ = 3.214; P = 0.0814] |
|  |  |  | Sex [F_(1,36)_ = 7.827; P = 0.0082] |
|  |  |  | Diet [F_(1,36)_ = 2.616; P = 0.1145] |
| 2C | LS glutamate content | 2-way ANOVA with Tukey’s multiple comparisons | Interaction [F_(1,34)_ = 4.696; P = 0.0373] |
|  |  |  | Sex [F_(1,34)_ = 7.796; P = 0.0085] |
|  |  |  | Diet [F_(1,34)_ = 8.037; P = 0.0077] |
| 2D | LS GABA content | 2-way ANOVA with Tukey’s multiple comparisons | Interaction [F_(1,36)_ = 0.9745; P = 0.3302] |
|  |  |  | Sex [F_(1,36)_ = 0.4434; P = 0.5097] |
|  |  |  | Diet [F_(1,36)_ = 7.051; P = 0.0117] |
| 3D | Δ Body weight (males) | 2-way ANOVA with Tukey’s multiple comparisons | Interaction [F_(2,63)_ = 16.76; P < 0.0001] |
|  |  |  | Treatment [F_(2,63)_ = 145.5; P < 0.0001] |
|  |  |  | Diet [F_(1,63)_ = 41.66; P < 0.0001] |
| 3E | Δ Body weight (females) | 2-way ANOVA with Tukey’s multiple comparisons | Interaction [F_(2,58)_ = 6.511; P = 0.0028] |
|  |  |  | Treatment [F_(2,58)_ = 87.22; P < 0.0001] |
|  |  |  | Diet [F_(1,58)_ = 115.6; P < 0.0001] |
| 4B | LS GLP-1R protein levels in males (band at 80 kDa) | 2-way ANOVA with Tukey’s multiple comparisons | Interaction [F_(2,24)_ = 1.042; P = 0.3683] |
|  |  |  | Treatment [F_(2,24)_ = 2.083; P = 0.1465] |
|  |  |  | Diet [F_(1,24)_ = 0.3694; P = 0.5490] |
| 4B | LS GLP-1R protein levels in females (band at 80 kDa) | 2-way ANOVA with Tukey’s multiple comparisons | Interaction [F_(2,24)_ = 0.5404; P = 0.5894] |
|  |  |  | Treatment [F_(2,24)_ = 1.127; P = 0.3406] |
|  |  |  | Diet [F_(1,24)_ = 0.7492; P = 0.3953] |
| 4C | LS GLP-1R protein levels in males (band at 53 kDa) | 2-way ANOVA with Tukey’s multiple comparisons | Interaction [F_(2,24)_ = 0.1324; P = 0.8766] |
|  |  |  | Treatment [F_(2,24)_ = 1.340; P = 0.2808] |
|  |  |  | Diet [F_(1,24)_ = 0.4244; P = 0.5210] |
| 4C | LS GLP-1R protein levels in females (band at 53 kDa) | 2-way ANOVA with Tukey’s multiple comparisons | Interaction [F_(2,24)_ = 0.4517; P = 0.6418] |
|  |  |  | Treatment [F_(2,24)_ = 1.842; P = 0.1803] |
|  |  |  | Diet [F_(1,24)_ = 3.548; P = 0.0718] |
| 5B | Total energy consumed in males at PND 63 | Mann–Whitney test. | P < 0.0001 |
| 5C | Total energy consumed in females at PND 63 | Mann–Whitney test. | P = 0.0185 |
| 6A | Total energy consumed in males at PND 75 | 2-way ANOVA with Tukey’s multiple comparisons | Interaction [F_(2,63)_ = 1.995; P = 0.1446] |
|  |  |  | Treatment [F_(2,63)_ = 8.548; P = 0.0005] |
|  |  |  | Diet [F_(1,63)_ = 1.231; P = 0.2715] |
| 6C | Total energy consumed in females at PND 75 | 2-way ANOVA with Tukey’s multiple comparisons | Interaction [F_(2,58)_ = 2.589; P = 0.0837] |
|  |  |  | Treatment [F_(2,58)_ = 19.80; P < 0.0001] |
|  |  |  | Diet [F_(1,58)_ = 0.4526; P = 0.5038] |
| Supp. Fig. 1A | Retroperitoneal fat tissue at PND 63 | 2-way ANOVA with Tukey’s multiple comparisons | Interaction [F_(1,98)_ = 19.82; P < 0.0001] |
|  |  |  | Sex [F_(1,98)_ = 95.17; P < 0.0001] |
|  |  |  | Diet [F_(1,98)_ = 180.0; P < 0.0001] |
| Supp. Fig. 1B | Gonadal fat tissue at PND 63 | 2-way ANOVA with Tukey’s multiple comparisons | Interaction [F_(1,100)_ = 0.1564; P = 0.6933] |
|  |  |  | Sex [F_(1,100)_ = 43.89; P < 0.0001] |
|  |  |  | Diet [F_(1,100)_ = 114.4; P < 0.0001] |
| Supp. Fig. 3A | Retroperitoneal fat tissue in males at PND 75 | 2-way ANOVA with Tukey’s multiple comparisons | Interaction [F_(2,62)_ = 2.470; P = 0.0928] |
|  |  |  | Treatment [F_(2,62)_ = 10.69; P = 0.0001] |
|  |  |  | Diet [F_(1,62)_ = 7.910; P = 0.0066] |
| Supp. Fig. 3B | Gonadal fat tissue in males at PND 75 | 2-way ANOVA with Tukey’s multiple comparisons | Interaction [F_(2,63)_ = 4.191; P = 0.0196] |
|  |  |  | Treatment [F_(2,63)_ = 13.71; P < 0.0001] |
|  |  |  | Diet [F_(1,63)_ = 7.281; P = 0.0089] |
| Supp. Fig. 3C | Retroperitoneal fat tissue in females at PND 75 | 2-way ANOVA with Tukey’s multiple comparisons | Interaction [F_(2,58)_ = 0.4527; P = 0.6381] |
|  |  |  | Treatment [F_(2,58)_ = 12.37; P < 0.0001] |
|  |  |  | Diet [F_(1,58)_ = 9.980; P = 0.0025] |
| Supp. Fig. 3D | Gonadal fat tissue in females at PND 75 | 2-way ANOVA with Tukey’s multiple comparisons | Interaction [F_(2,58)_ = 0.4605; P = 0.6332] |
|  |  |  | Treatment [F_(2,58)_ = 6.095; P = 0.0040] |
|  |  |  | Diet [F_(1,58)_ = 12.70; P = 0.0007] |
| Supp. Fig. 4B | Food intake in males at PND 63 | 2-way ANOVA with Tukey’s multiple comparisons | Interaction [F_(1,134)_ = 11.13; P = 0.0011] |
|  |  |  | Group [F_(1,134)_ = 11.75; P = 0.0008] |
|  |  |  | Diet [F_(1,134)_ = 12.47; P = 0.0006] |
| Supp. Fig. 4C | Food intake in females at PND 63 | 2-way ANOVA with Tukey’s multiple comparisons | Interaction [F_(1,123)_ = 0.5773; P = 0.4488] |
|  |  |  | Group [F_(1,123)_ = 2.110; P = 0.1489] |
|  |  |  | Diet [F_(1,123)_ = 5.780; P = 0.0177] |
| Supp. Fig. 4D | Food intake in males at PND 75 | 2-way ANOVA with Tukey’s multiple comparisons | Interaction [F_(5,126)_ = 3.785; P = 0.0032] |
|  |  |  | Group [F_(5,126)_ = 3.360; P = 0.0070] |
|  |  |  | Diet [F_(1,126)_ = 20.90; P < 0.0001] |
| Supp. Fig. 4E | Food intake in females at PND 75 | 2-way ANOVA with Tukey’s multiple comparisons | Interaction [F_(5,116)_ = 6.954; P < 0.0001] |
|  |  |  | Group [F_(5,116)_ = 2.874; P = 0.0175] |
|  |  |  | Diet [F_(1,116)_ = 55.35; P < 0.0001] |
